# Nonparametric Test for Volatility in Clustered Multiple Time Series

**DOI:** 10.1007/s10614-023-10362-x

**Published:** 2023-03-16

**Authors:** Erniel B. Barrios, Paolo Victor T. Redondo

**Affiliations:** 1 Monash University Malaysia, Selangor, Malaysia; 2 King Abdullah University of Science and Technology, Thuwal, Saudi Arabia

**Keywords:** Multiple time series, Volatility, Clustering, Nonparametric test, Sieve bootstrap

## Abstract

Contagion arising from clustering of multiple time series like those in the stock market indicators can further complicate the nature of volatility, rendering a parametric test (relying on asymptotic distribution) to suffer from issues on size and power. We propose a test on volatility based on the bootstrap method for multiple time series, intended to account for possible presence of contagion effect. While the test is fairly robust to distributional assumptions, it depends on the nature of volatility. The test is correctly sized even in cases where the time series are almost nonstationary (i.e., autocorrelation coefficient $\approx 1$). The test is also powerful specially when the time series are stationary in mean and that volatility are contained only in fewer clusters. We illustrate the method in global stock prices data.

## Introduction

With increasing storage space availability, real-time stock prices can now be recorded, including short-run spikes triggered by random shocks, e.g., news on a revamp of the management of a company, policy adjustment. This may not be an issue if stock prices are recorded on lower frequencies (e.g., weekly or monthly) since there will be a natural smoothing of irregular movements resulting from aggregation or averaging from higher to lower frequencies. As a result, low frequency measurements can lead to losses in valuable information about prices hence, these are typically recorded at the real-time level or at least at some intra-day levels. This could trigger high frequency time series data to manifest such shocks as conditional heteroskedasticity (volatility). Furthermore, individual securities may behave independently, but contagion within a sector, a market, a region, or even globally can force a group or all securities to exhibit similar price movement patterns.

Public information shared among the players in the financial market along with the impact of regulatory agencies may drive behavior of securities in the market. Different stakeholder may utilize the public information differently from others, resulting to a stylized behavior of stock prices. It is then imperative for both the regulatory agencies and stock market players to assess the spillover effects of certain news in a company or on the sector as whole as certain policy options or investment strategies can be developed depending on how the market reacts to this information. Given high frequency time series data, this phenomenon is viewed as volatility in clustered multiple time series data, i.e., while each time series (e.g., prices of different securities) behave independently, the events associated with public information available drives the time series into different paths, but a common pattern of the movement is taken as the common volatility pattern among multiple time series data.

Modeling procedures are available for multiple and multivariate time series data, but these are anchored on distributional assumptions about the random shocks and are greatly affected by irregularities or stylized facts about the data like volatility. Volatility causes perturbation in the dynamic behavior of the process, resulting to more complex data generating process causing difficulty in estimation and produces chaotic forecasts. Volatility has the potential to divert forecasts away from the direction of the time series even after the effect of localized perturbation vanished.

The paper is organized as follows: Sect. 2 summarizes previous literature on multiple time series and volatility; Sect. 3 presents the estimation algorithm and the proposed test for volatility in clustered time series; Sect. 4 discusses results of simulation studies to illustrate the size and power of the proposed test; Sect. 5 presents the application of the test to actual data; Sect. 6 summarizes the conclusions.

## Multiple Time Series, Volatility and Contagion

Arellano and Bond (1991) considered multiple time series data as panel data whose common autoregressive parameter and random effect of individual time series were estimated using generalized method of moments (GMM). Although the method usually fails to converge when the length of time series is larger than the number of time series in the panel, Veron Cruz and Barrios (2014) proposed an estimation procedure that incorporates maximum likelihood estimation (MLE) and best linear unbiased predictors (BLUP) into the backfitting algorithm. Veron Cruz and Barrios (2014) noted that the advantages of the method are affected by the variance of the error term (possibly by heteroskedasticity), and to address this problem, Ramos et al (2016) proposed an estimation procedure that is robust to conditional heteroskedasticity of the multiple time series. Even in the presence of volatility, Ramos et al (2016) observed improvement in parameter estimates as well as the predictive ability of the fitted model. This is however still affected by localized non-stationarity induced by the block bootstrap method even if there is really no global heteroskedasticity in the time series. Veron Cruz and Barrios (2014) and Ramos et al (2016) are the basis for the postulated multiple time series presented in Sect. 3.

Volatility in time series has been typically assessed by incorporating models for conditional heteroscedasticity into the model structure, e.g., autoregressive conditional heteroskedastic (ARCH) model Engle (1982), generalized autoregressive conditional heteroskedastic (GARCH), Bollerslev (1986). Other volatility models which address issues regarding ARCH- and GARCH-like violation of the non-negative constraints for the variances are also proposed, e.g., exponential generalized autoregressive conditional heteroskedastic model (EGARCH), Nelson (1991). But these more general models also encounter issues such as in estimation (due to complex likelihood functions) and in forecasting (since volatility drive forecast errors to explode). While volatility can be generalized to any of these models, ARCH (1) was used in Sect. 3. The algorithm presented in Sect. 3 can be modified minimally to consider a more general volatility model.

The literature on multiple time series and volatility (separately) has been extensive, but a test for the presence of volatility in multiple time series is still open. This viewpoint of volatility in a clustered setting can help explain the dynamics of contagion in phenomena governed by certain policies or regulations like the financial markets.

Contagion is a phenomenon that is closely intertwined into multiple time series and volatility. Contagion in multiple time series results from availability of high frequency data (e.g., intra-day) since latent volatility characteristics can actually be inferred from realized volatility (see for example McAleen and Medeiros, 2008). Financial contagion representing the spillover effect of shocks that fuels systemic risk in an economy. Vodenska and Becker (2019) emphasized the need to understand the network structure of global financial markets, this will provide the stakeholders some insights on the repercussions of shock into the economy and subsequent mitigation strategies for the risks can be identified. Extreme events (tail risk) contagion in the financial markets as a result of the COVID-19 pandemic was studied by Guo et al. (2021) using some indices, these are then used in characterizing the contagion channels in the financial markets.

## Test for Volatility in Multiple Time Series

Knowledge on whether volatility is present or not in the time series offers an opportunity to better understand the dynamic behavior of the data, thus facilitating modeling and forecasting. Campano and Barrios (2011) proposed a robust estimation procedure for time series data that exhibit structural change. Furthermore, Campano (2012) proposed a test for volatility in a time series data. Predictive ability of estimated models during tranquil period can be enhanced resulting from robust estimation of the model, noted Campano and Barrios (2011). The aim of this paper is to develop a nonparametric test of volatility in a possibly clustered multiple time series data. Clustering in multiple time series occurs from the simultaneous jumps (co-jumps) in prices associated with major news affecting a particular sector, see for example Caporin et al. (2017).

Given $N$ time series each with $T$ observations, Veron Cruz and Barrios (2014) considered the following model:

1 $$ Y_{i,t} = \phi Y_{i,t - 1} + \lambda_{i} + u_{i,t} ,\;\;\lambda_{i} \sim \left( {\mu_{i} ,\sigma_{\lambda }^{2} } \right)\;\;u_{i,t} \sim \left( {0,\sigma_{u}^{2} } \right) $$

for $i=\mathrm{1,2},\dots ,N$ and $t=\mathrm{1,2},\dots ,T$. In a telecommunication setting, ${Y}_{i,t}$ can be the data usage of the ${i}^{th}$ customer for time t, and ${\lambda }_{i}$ can be the person-specific variability in the data usage while ${u}_{i,t}$ can represent the random shock that exhibit similar distributional properties among all subscribers over time. Suppose that the N time series is grouped into the m clusters, each with ${n}_{j}$ elements, $N={n}_{1}+\dots +{n}_{m}$. Model (1) is modified to account for clustered conditional heteroscedasticity in the error term ${u}_{i,t}$ as follows:

2 $$ Y_{i,t} = \phi Y_{i,t - 1} + \lambda_{i} + u_{i,t} ,\;\;\lambda_{i} \sim \left( {\mu_{i} ,\sigma_{\lambda }^{2} } \right),\;\;u_{i,t} = v_{t} \sigma_{kt} ,\;\;v_{t} \sim N\left( {0,1} \right) $$

where ${\sigma }_{kt}^{2}$ accounts for conditional heteroscedasticity present in cluster $k$, $k=1,\dots ,m$ and ${v}_{t}$ is a white noise process. This implies that the time series within each cluster exhibit similar volatility behavior, and volatility models may possibly vary across different clusters.

Now, assume that the volatility model for each cluster is ARCH(1), and that the time series within the cluster $\left({n}_{k}\right)$ share the same set of parameters. Also, suppose the common dependence structure of the series is captured by the same parameter $\phi$ in the dynamic model while the series-specific variation is expressed through the random effects (${\lambda }_{i}$) for each time series. Clearly, there is a need to estimate the parameters shared globally across all the time series, the parameters of the volatility model shared within the cluster, and series-specific random effects.

### Estimation Phase

Here, model (2) is estimated in an iterative algorithm based on the backfitting framework. The backfitting algorithm estimates each component sequentially through the partial residuals obtained whenever a component has been estimated. Even though initially ignoring some components introduces bias in the estimates, such bias decreases in the iteration of implementing the algorithm. For model (2), we assume three components to be estimated separately, namely, the common autoregressive parameter $\phi$, the random effects ${\lambda }_{i}$ and the ARCH(1) parameters per cluster. The initial estimates for ${\lambda }_{i}$ are obtained by ignoring the autoregressive and error terms from the model. On the other hand, the parameter $\phi$ is initialized by fitting the residuals after removing the first estimated component (${\widehat{\lambda }}_{i}$). Meanwhile, the volatility parameters are initialized with the residuals obtained after removing the first and second estimated components (${\widehat{\lambda }}_{i}$ and $\widehat{\phi }$). Specifically, for the ${b}^{th}$ iteration:

1. Given previous estimates $${\widehat{\phi }}^{(b-1)}$$ and computed residuals $${r}_{i,t}^{**\left(b-1\right)}={Y}_{i,t}-{\widehat{\phi }}^{\left(b-1\right)}{Y}_{i,t-1}$$, estimate the series-specific random effects from the residuals $${r}_{i,t}^{**\left(b-1\right)}$$ using the BLUP method, i.e., $${\widehat{\lambda }}_{i}^{(b)}={\widehat{\mu }}_{i}$$ since $$E\left({\lambda }_{i}\right)={\mu }_{i}$$.
2. Compute new residuals:${r}_{i,t}^{*\left(b\right)}={Y}_{i,t}-{\widehat{\lambda }}_{i}^{(b)}$.
  Rescaling of residuals ${r}_{it}^{*\left(b\right)}$ by the estimated volatility component ${\widehat{\sigma }}_{it}^{2}$ is not necessary since the backfitting algorithm is fairly optimal with additivity of the model, see for example Opsomer (2000).
3. Estimate $\phi$ by ${\widehat{\phi }}^{(b)}$ from the following bootstrap method sub-steps:
  - For each of the N time series of residuals ${r}_{it}^{*\left(b\right)}$, estimate $\phi$ as the autoregressive parameter and intercept of the residuals using conditional least squares to obtain ${\widehat{\phi }}_{i}$.
  - Resample from ${\widehat{\phi }}_{i}, i=1,\dots ,N$, to obtain ${\widehat{\phi }}^{(b)}= {\widehat{\phi }}^{BS(b)}$ (simple random sample with replacement of size $N$, for $R$ replicates). This is an ordinary bootstrap since each time series $i$ provided one estimate for the autoregressive parameter $\left({\widehat{\phi }}_{i}\right)$.
4. Compute two forms of new residuals:
  $$ Y_{i,t} - \hat{\phi }^{\left( b \right)} Y_{i,t - 1} \;\;{\text{and}}\;\;r_{it}^{***\left( b \right)} = Y_{i,t} - \hat{\lambda }_{i}^{\left( b \right)} - \hat{\phi }^{\left( b \right)} Y_{i,t - 1} $$
  The first form of residuals $${r}_{it}^{**\left(b\right)}$$ will be used to estimate the series-specific effects $${\lambda }_{i}$$ in the next iteration while the second form of residuals $${r}_{it}^{***\left(b\right)}$$ will be used to estimate the volatility model. Note that the bootstrap intercept is also subtracted from the original observations $${Y}_{i,t}$$ in the second form of residuals $${r}_{i,t}^{***\left(b\right)}$$. Presence of volatility in the model affects the level of the residuals, and by subtracting the bootstrap intercept, stabilization in the levels of the random component is achieved.
5. For each time point $$(t)$$, the second form of residuals $${r}_{i,t}^{***(b)}$$ mimics the behavior of the random shocks $${u}_{i,t}$$. Thus, we use the square of these residuals, $${\widehat{\sigma }}_{i,t}^{2}= {\widehat{u}}_{i,t}^{2}={\left({r}_{i,t}^{***(b)}\right)}^{2}$$, as an unbiased estimator of the heteroscedastic variance $${\sigma }_{i,t}^{2}$$ and the squared shocks $${u}_{i,t}^{2}$$. Estimate the variance model, e.g., $$\left({\sigma }_{i,t}^{2}\right)={\alpha }_{k,0}+{\alpha }_{k,1}{u}_{i,t-1}^{2}$$ (for ARCH (1)) using $$\left({\widehat{u}}_{i,t}^{2}, {\widehat{u}}_{i,t-1}^{2}\right)$$ through ordinary least squares (OLS) estimation to obtain $${\widehat{\alpha }}_{0i}^{(b)}$$ and $${\widehat{\alpha }}_{1i}^{(b)}$$ for each series.
6. For cluster $k$, estimate the volatility parameters ${\alpha }_{k,0}$ by ${\widehat{\alpha }}_{k,0}^{(b)}$ (the mean of ${\widehat{\alpha }}_{0i}^{(b)}$, $i = 1,\dots , {n}_{k}$) and ${\alpha }_{k,1}$ by ${\widehat{\alpha }}_{k,1}^{(b)}$ (the mean of ${\widehat{\alpha }}_{1i}^{(b)}$ $i = 1,\dots , {n}_{k}$). Here, ${\widehat{\alpha }}_{0i}^{(0)}$ and ${\widehat{\alpha }}_{1i}^{(b)}$ are the individual ARCH (1) parameter estimates of ${i}^{th}$ time series. This implies that different ARCH(1) parameters are estimated for each cluster.

Then, we iterate from Step 1 until convergence, e.g., when parameter changes in-between iteration by less than the tolerance level $\varepsilon$.

### Testing for Volatility

Given parameter estimates from the Estimation Phase,

1. Reconstruct variance components for each resample through
  $$ \left( {\hat{\sigma }_{i,t}^{2} } \right) = \hat{\alpha }_{k,0} + \hat{\alpha }_{k,1} \hat{u}_{i,t - 1}^{2} . $$
2. Generate $${u}_{i,t}^{*}$$ from $$N\left(0,{\widehat{\sigma }}_{i,t}^{2}\right)$$.
3. Compute replicates of ${Y}_{it}$ as ${Y}_{i,t}^{*}=\widehat{\phi }{Y}_{i,t-1}^{*}+{\widehat{\lambda }}_{i}+{u}_{i,t}^{*}$
4. Estimate parameters from each replicate of the data using the Estimation Phase presented above.

The proposed algorithm is based on the sieve bootstrap, see for example, Buhlmann (1997). The algorithm derives the empirical distribution of the parameters in the model through a sieve bootstrap method, the distribution is then used in making decisions pertaining to the hypothesis being tested.

Multiple clusters are tested simultaneously. To control the familywise error rate (FWER), size $\alpha$ of the test is adjusted to $\alpha /m$ (Bonferroni correction) where $m$ is the number of clusters, see for example, Wright (1992). Given the bootstrap replicates, ${\left(\frac{\alpha }{2m}\right)}^{th}$ and ${\left(1-\frac{\alpha }{2m}\right)}^{th}$ percentiles of ${\widehat{\alpha }}_{k,1}$ s is computed and are used to test the significance of the parameter estimate for each cluster. Non-inclusion of zero in the interval provides enough empirical evidence against the null hypothesis (i.e., no significant volatility) while inclusion of zero indicates no evidence against the null hypothesis. For the variance model, ${\alpha }_{k,1}=0$ indicates no volatility (assuming ARCH (1) model). Hence, the test is equivalent to the null which is absence of volatility of specific model, e.g., ARCH (1) again the alternative that volatility of specific model exists.

The method discussed above assumes that clusters are identified. Existence of clusters (number of clusters and membership of time series to a cluster) can be postulated by the analyst, e.g., stocks that are more likely involved in a possible contagion. Alternatively, number and cluster membership can be determined statistically through time series clustering, see for example, Aghabozorgi et al (2015).

## Simulation Study

We designed a simulation study to investigate the computational optimality of the test. Some conditions about the data generating process are controlled, and this includes: number of time series (N = 50), length of each time series (T = 50); autoregressive parameter ($\phi$=0.6, 0.95 to represent stationary and near nonstationary time series, respectively); mean of random effect (${\mu }_{i}=0$); constant standard deviation of random effect across all time series; number of clusters (1 or 5, absence or presence of clustering, respectively); ARCH parameters [$\left({\alpha }_{k0}=1, {\alpha }_{k1}=1\right)$-presence of volatility, $\left({\alpha }_{k0}=1, {\alpha }_{k1}=0\right)$-absence of volatility]; and when there are 5 clusters, 1 or 3 of the clusters are set to exhibit an ARCH(1) type of volatility. In all cases, level of significance is set at $\left(\alpha =0.05\right).$

The data was simulated with Eq. (2) as the data generating process. Random variables are first generated from the corresponding distribution. The white noise process ${v}_{t}$ was generated from the standard normal distribution. After initialization of the time series, repetitive substitution of previous values, assumed parameters, current and past values of random components to Eq. (2) is done until 2 T time points are generated. The first half of the simulated time series are dropped as this might have been influenced by initial values.

The nonparametric test is compared to a parametric test based on ARCH (1) model where each time series is treated in a univariate context. The parametric test for volatility is based on the likelihood ratio test, see for example, Engle (1982). The goal of the comparison is to assess whether knowledge of clustering can contribute in detecting group volatility. Power and size comparisons between parametric and nonparametric tests for various scenarios are summarized in Table 1.

**Table 1:** Simulation results for scenarios without misclassified time series in a cluster

Scenario	Autoregressive parameter ($\phi$)	Power of the test		Size of the test	
		Nonparametric	Parametric	Nonparametric	Parametric
Single Cluster	0.6	1.0000	0.3762	0.0117	0.0224
Single Cluster	0.95	0.9081	0.2193	0.0000	0.0681
5 Clusters, Only 1 Cluster with Volatility	0.6	0.6250	0.4110	0.0078	0.0236
5 Clusters, Only 1 Cluster with Volatility	0.95	0.2711	0.2410	0.0042	0.0648
5 Clusters, With 3 Clusters with Volatility	0.6	0.5854	0.3852	0.0061	0.0213
5 Clusters, With 3 Clusters with Volatility	0.95	0.1383	0.2288	0.0000	0.0600

### Single Cluster, No Volatility

If all time series forms a single cluster, the nonparametric test is correctly-sized regardless on whether the time series are stationary (in mean) or nearly non-stationary. The parametric test is also correctly-sized when the time series is stationary in mean. However, size of the parametric test is distorted when the time series approaches nonstationarity in mean. This is not the case in the nonparametric test since all replicates under near nonstationarity failed to reject the null hypothesis of no volatility.

### Single Cluster, Volatility (ARCH) is Present

ARCH-type volatility model is induced to the simulated time series in cases where there is only a single cluster. The nonparametric test that considers all time series to provide evidence against the null hypothesis of no volatility yield very high power compared to the parametric counterpart that considers each time series individually, regardless of the state of stationarity in mean. In cases where the time series are stationary in mean, the nonparametric test was able to provide evidence against the null hypothesis for all replicates of the simulated data, while very low power was observed in the parametric test. As the time series approaches nonstationarity, both the nonparametric and parametric tests suffer a decline in power, but the decline in power of the parametric test is much larger than the decline in power of the nonparametric test (still exhibiting a reasonable power).

### 5 Clusters, Volatility (ARCH) is Present in 1 Cluster

Assuming 5 clusters, without inducing volatility in the simulated time series, both parametric and nonparametric test are correctly-sized. However, when the time series approaches nonstationarity, the parametric test already suffers from size distortion since the procedure relies heavily on the stationarity in mean assumption. This is not the case for the nonparametric test that is still correctly-sized even if the time series approaches near-nonstationarity. When volatility is induced in simulated time series in one cluster (time series in four other clusters do not contain volatility), the nonparametric test exhibit over 20% advantage in power compared to the parametric test in time series that are stationary in mean. When the time series approaches nonstationarity, both the parametric and nonparametric tests have lower power, the nonparametric test though still have relative advantage over the parametric test.

### 5 Clusters, Volatility (ARCH) is Present in 3 of the Clusters

Both the parametric and nonparametric tests are consistently correctly-sized when all the time series in 5 clusters exhibit stationarity in mean. The parametric test however, exhibit distortion in size when the time series in all clusters approaches nonstationarity in mean, this is not the case for the nonparametric test which is still correctly sized even when the time series approaches nonstationarity. As volatility is induced in three of the five clusters, the nonparametric test still has over 20% advantage in terms of power over the parametric test. Power of both parametric and nonparametric tests suffer as the time series across all clusters approaches nonstationarity.

### Misclassified Time Series

To verify robustness of the test to possible misclassification of time series into a cluster, a cluster of 50 time series with volatility is deliberately contaminated with some time series that does not exhibit volatility. Furthermore, similar cluster of 50 time series without volatility contaminated with some time series that actually exhibit volatility.

With 50 time series simulated to exhibit volatility, one time series (2%) or five (10%) time series that does not exhibit volatility were included. Provided that the time series are stationary (autoregressive parameter of 0.60), the test is able to identify volatility for all replicates. Relatively lower power (80%) is obtained when autoregressive parameter is 0.95.

The test is still able to detect even with only one (2%) or five (10%) time series with volatility are induced in a cluster of 50 time series. The chance of detecting volatility increases with more time series that actually exhibit volatilities in a cluster. Thus, regardless of the actual number of time series that exhibits volatility, the test is capable of detection of such. See Table 2 for details.

**Table 2:** Simulation results for scenarios with misclassified time series in a cluster

Scenario	Autoregressive parameter ($\phi$)	$P\left(\mathrm{Rejecting} {H}_{0}\right)$
No Volatility (2% with Volatility)	0.60	0.1162
No Volatility (10% with Volatility)	0.60	0.4731
No Volatility (2% with Volatility)	0.95	0.2062
No Volatility (10% with Volatility)	0.95	0.2513
With Volatility (2% No Volatility)	0.60	1.0000
With Volatility (10% No Volatility)	0.60	1.0000
With Volatility (2% No Volatility)	0.95	0.8077
With Volatility (10% No Volatility)	0.95	0.7913

## Application in Stock Market Price Indices

Contagion is a common event in stock markets usually resulting from interdependence among securities and among stock brokers. Volatility is another stylized fact among indicators that characterizes behavior of the market, often monitored at very high frequencies by various stakeholders. Lyocsa and Horvath (2018) noted that there is evidence of contagion from the US stock market to Japan, United Kingdom, France, Germany, Hong Kong, and Canada. They further noted that contagion is not just a crisis-specific event, but is present in the market all the time. Dewandaru et al. (2018) further observed that during the major crisis in European equity markets, contagion effects generated short-term shocks, also noted that there is evidence that the most recent US subprime crisis is brought about by contagion effect. These short-term shocks can easily drive volatility of key market indicators like prices.

We used prices of stocks traded in the European and US markets to investigate presence of volatility associated with contagion effect. Regional contagion can cause volatility among stock market prices in the region. In understanding the dynamic behavior of stock prices, time series data of prices of 30 stocks are postulated to cluster into European (19 stocks) and US (11 stocks) regions. Daily prices during 2011–2016 period are used in the analysis. The European and US markets and the period 2011–2016 were selected because of the contagion reported in the literature in these regions within the period. All stocks prices with available data from Yahoo Finance (https://finance.yahoo.com/quote/DATA.L/history?p=DATA.L) are include for the illustration discussed in this section.

### Original Time Series Data

**Fig. 1:**
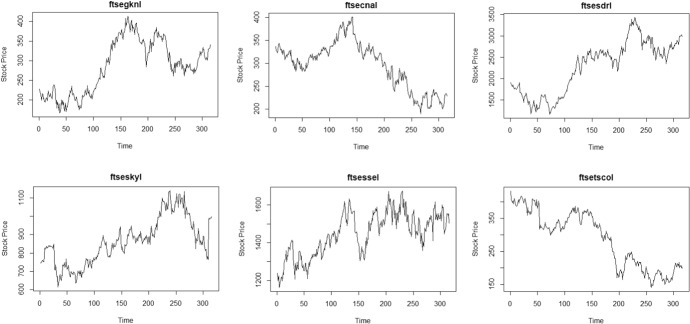
Time plot of some European stock prices

**Fig. 2:**
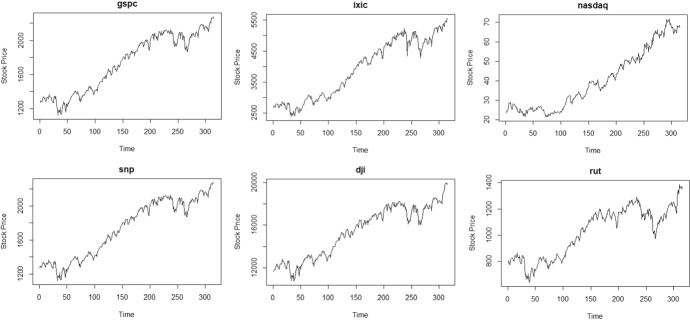
Time plot of some US stock prices

Six of nineteen European stocks are plotted in Fig. 1, while six of the eleven stocks in the US market are plotted in Fig. 2. While there are some periods where volatility seems to exists, this can potentially be masked by overall nonstationarity. From Table 3, The original time series data both from the European and US markets exhibit nonstationarity, most of the estimated autoregressive parameters are 0.99 or 0.98, smallest value was in a stock in the US market where autoregressive parameter is 0. 937.With the original time series data, nonstationarity in mean is dominating, so that the parametric test for volatility failed to reject the null hypothesis of no volatility for all time series, see Table 3 for details.

**Table 3:** Univariate analysis of 30 time series data

Stocks	Cluster	AR (1) Estimate	*p* value of parametric test assuming ARCH (1)
gdaxi	Europe	0.991521	0.452747
ftseanthl	Europe	nonstationary	–
ftseantoi	Europe	0.987608	0.211045
ftsebal	Europe	0.992843	0.755044
ftsebatsl	Europe	0.993605	0.895994
ftsegknl	Europe	0.985435	0.148366
ftsecnal	Europe	0.986601	0.715972
ftsepfgl	Europe	0.996475	0.247612
ftsepnsl	Europe	0.995587	0.965042
ftseprul	Europe	0.993537	0.639695
ftserbl	Europe	0.996969	0.729532
ftserrl	Europe	0.987278	0.97215
ftsesdrl	Europe	0.990858	0.052396
ftseshpl	Europe	0.992136	0.189234
ftseskyl	Europe	0.973691	0.838058
ftsessel	Europe	0.951328	0.303283
ftsestjl	Europe	0.996451	0.767607
ftsetscol	Europe	0.994495	0.549249
ftsevodl	Europe	0.989421	0.937241
gspc	US	0.997792	0.66884
ixic	US	0.997112	1.19E-07
nasdaq	US	0.998013	0.284526
nya	US	0.990934	0.149703
rut	US	0.993939	0.508075
snp	US	0.997792	0.66884
ta	US	0.969655	0.829248
tsx	US	0.956174	0.17366
xax	US	0.937966	0.912286
bvsp	US	0.972485	0.082685
dji	US	0.996645	0.421252

Using the estimation procedure for clustered time series data described in Sect. 3, parameters of the mean and variance models are estimated per cluster and presented in Table 4. In the multiple time series framework, we assumed similar model for the mean of the time series. The common autoregressive parameter is estimated at 0.9863, which is within the values of estimated autoregressive parameters (univariate) for the individual time series in Table 3.

**Table 4:** Estimate of the common autoregressive parameter and the ARCH (1) parameters per region

Autoregressive Parameter ($\widehat{\phi }$)	Volatility Slope Coefficient ($\widehat{\mathrm{\alpha }}$_k,1_)	
	European	US
0.9863	2.980 (0.4245,3.4143)*	− 0.021 (− 0.1476, 0.1731)*

^*^Bonferroni corrected 95% Confidence Interval

The Bonferroni corrected CI for the European market do not include 0, indicating that as a cluster, the European market exhibits volatility. Note that consistent with results of simulation studies, the parametric test failed to provide empirical evidence on the existence of volatility, while the nonparametric test was able to recognize empirical evidence of joint volatility (possibly caused by contagion) among the stocks in the European market. The Bonferroni corrected CI for the US market includes 0, hence, even the nonparametric test failed to recognize empirical evidence of the existence of group volatility among the stocks in the US market. Power of the nonparametric test diminish when the time series are nearly nonstationary.

### First Differenced Time Series

**Fig. 3:**
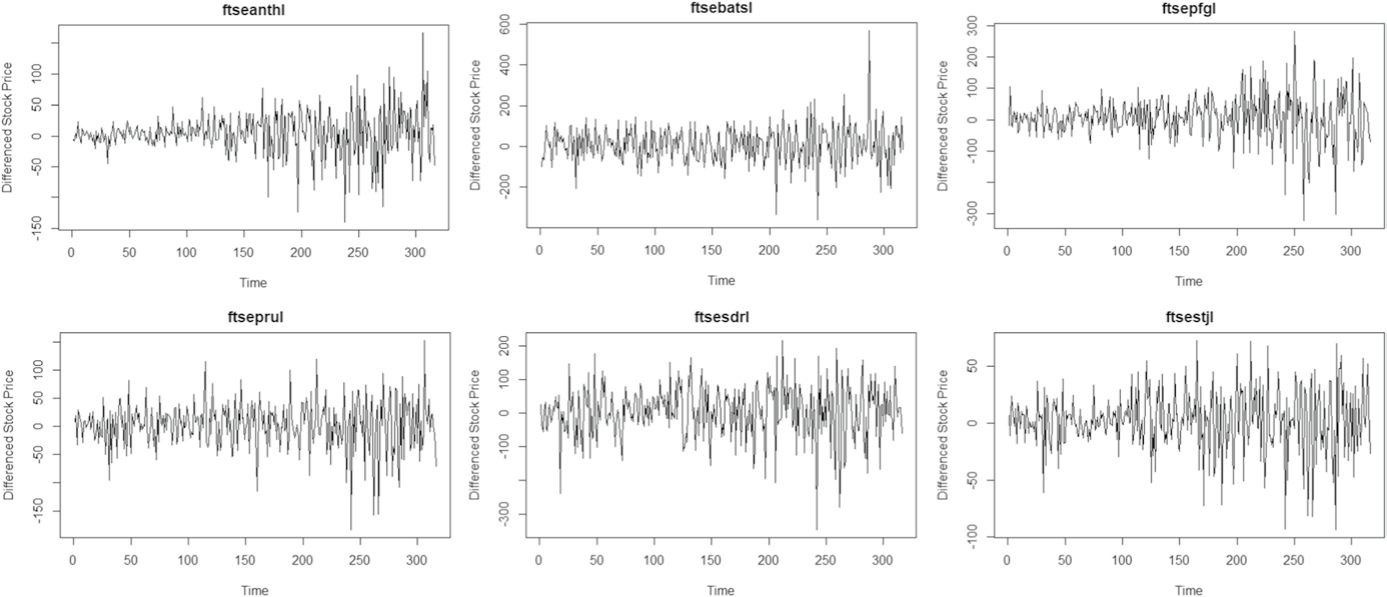
Time plot of some first differenced European stock Prices

**Fig. 4:**
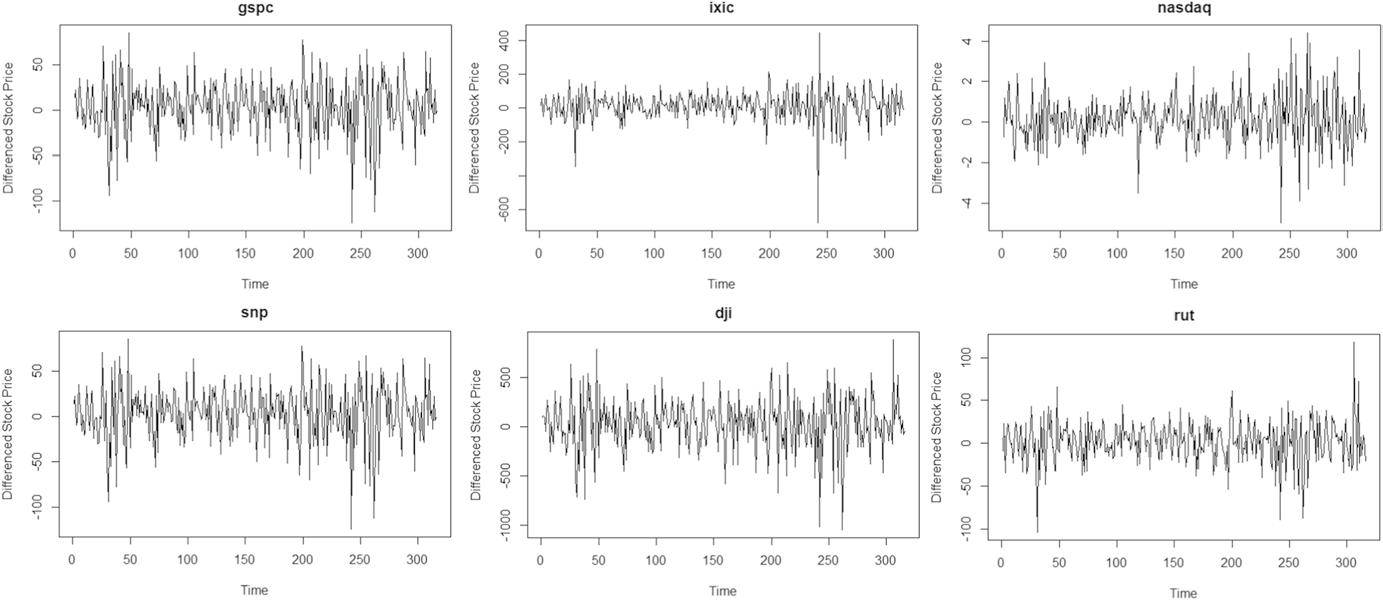
Time plot of some first differenced US stock prices

The parametric test for volatility suffers from size distortion when the time series approaches nonstationarity, which is not the case in the nonparametric test. Also, power is reduced even in the nonparametric test as the time series approaches nonstationarity, but with greater reduction in power for the parametric test. First differences of the time series are obtained to mitigate presence of nonstationarity. Time plots of six stocks in the European market are given in Fig. 3 and the time plots of six stocks in the US market are given in Fig. 4. Both clusters now exhibit stationary behavior and volatility has become more visually evident.

Univariate analysis was done with the individual (first-differenced) time series, estimates and results of parametric tests for volatility are summarized in Table 5. All first differenced time series are now stationary. In fact, many of the time series are actually random walk since no dependence structure is evident from the first differenced time series. Only four stocks in the European market and two stocks in the US market still exhibit dependencies after first differencing. The parametric test for volatility identifies only one time series in the European and one in the US market to exhibit volatility.

**Table 5:** Univariate analysis of 30 time series data (First Differenced)

Stocks	Cluster	AR (1) Coefficient		*p* value of parametric test assuming ARCH (1)
		Estimate	*p* value	
gdaxi	Europe	− 0.07667	0.171013	0.484642
ftseanthl	Europe	− 0.15284	0.006077	0.962022
ftseantoi	Europe	− 0.10686	0.059935	0.363237
ftsebal	Europe	− 0.10563	0.059022	0.625738
ftsebatsl	Europe	− 0.01579	0.779105	0.965093
ftsegknl	Europe	− 0.17087	0.002034	0.459401
ftsecnal	Europe	− 0.07678	0.170875	0.726868
ftsepfgl	Europe	− 0.04521	0.421263	0.255153
ftsepnsl	Europe	− 0.11653	0.037212	0.821268
ftseprul	Europe	− 0.0874	0.120066	0.632106
ftserbl	Europe	− 0.08967	0.109265	0.598744
ftserrl	Europe	− 0.03911	0.486438	0.980128
ftsesdrl	Europe	− 0.02777	0.621233	0.052873
ftseshpl	Europe	− 0.03354	0.552309	0.24739
ftseskyl	Europe	− 0.08359	0.135394	0.747185
ftsessel	Europe	− 0.1061	0.058469	0.275747
ftsestjl	Europe	− 0.1211	0.030172	0.877905
ftsetscol	Europe	− 0.05697	0.31021	0.769745
ftsevodl	Europe	− 0.00777	0.890174	0.936011
gspc	US	− 0.0936	0.094255	0.604614
ixic	US	− 0.17454	0.0016	0.008302
nasdaq	US	− 0.14845	0.007587	0.975516
nya	US	− 0.10032	0.072651	0.401013
rut	US	− 0.04546	0.418536	0.479858
snp	US	− 0.0936	0.094255	0.604614
ta	US	0.032897	0.55791	0.899131
tsx	US	− 0.08347	0.136433	0.377397
xax	US	− 0.08884	0.113139	0.608238
bvsp	US	− 0.01567	0.780449	0.110908
dji	US	− 0.09195	0.100237	0.360457

We also used the estimation procedure for clustered data described in Sect. 3 for the first differenced time series. Parameters of the mean and variance models are estimated per cluster and presented in Table 6. From the multiple time series assumption, the common autoregressive parameter is estimated to be − 0.0809, within the range of values of the autoregressive coefficients from the univariate analysis in Table 5.

**Table 6:** Estimate of the common autoregressive parameter and the ARCH (1) Parameters per region (First Differenced)

Autoregressive Parameter ($\widehat{\phi }$)	Volatility Slope Coefficient ($\widehat{\mathrm{\alpha }}$_k,1_)	
	European	US
-0.0809	33.85 (1.2952, 27.4435)*	1.02 (0.8197, 1.5156)*

^*^Bonferroni corrected 95% Confidence Interval

From Table 6, the Bonferroni corrected CI for the European market do not include 0, indicating that as a cluster, the European market exhibits volatility. Similar is true for the US market, the Bonferroni corrected CI also precludes zero, indicating presence of volatility among the clustered time series. Recall that the simulation study indicates higher power for the nonparametric test when the individual time series are stationary. While the parametric test for volatility in Table 5 identifies only one time series to exhibit volatility, the nonparametric test in Table 6 provides empirical evidence that clustered volatility is present in both the European and US markets.

## Conclusions

Given clustered time series data, a nonparametric test for volatility is proposed, this accounts for the possible contagion effect among time series in the same cluster. The simulation study illustrate that the test is correctly-sized even when the multiple time series approaches nonstationarity. The test is powerful if volatility is contained in fewer clusters only, a resemblance of localized contagion effect. As contagion causing volatility become global in nature, i.e., as more clusters are affected by volatility, even the nonparametric test exhibits low power. Note however that widespread volatility, i.e., practically all time series manifest volatility behavior, is also the case where volatility often becomes more obvious even visually. The nonparametric test offers a method of testing volatility in multiple time series that exhibit clustering, and that volatility spillover is contained only in few clusters. In the presence of contagion, whether local or global, the nonparametric test can benefit from the simultaneous evidence that all time series can provide against absence of volatility. A clear understanding of presence of volatility will facilitate identification and estimation of models that can generate reliable forecast of indicators involved, hence, better risk management in sectors that manifest such volatile behavior like the financial markets.

A more general abstraction of volatility in clustered multiple time exhibiting a generalized behavior can further enhance tools that could better understand features of some complicated phenomenon.

## Data Availability

https://github.com/ptredondo/NonparVolTest.
